# Mycovirus-Like DNA Virus Sequences from Cattle Serum and Human Brain and Serum Samples from Multiple Sclerosis Patients

**DOI:** 10.1128/genomeA.00848-14

**Published:** 2014-08-28

**Authors:** Iranzu Lamberto, Karin Gunst, Hermann Müller, Harald zur Hausen, Ethel-Michele de Villiers

**Affiliations:** a Division for the Characterization of Tumorviruses, Deutsches Krebsforschungszentrum, Heidelberg, Germany; b Institute for Virology, Faculty of Veterinary Medicine, University of Leipzig, Leipzig, Germany

## Abstract

Myco-like viruses have been isolated from fungi, feces of various animals, and plant leaves. We report here the isolation of 3 complete genome sequences of gemycircularvirus-related viruses from healthy bovine serum and human brain and serum samples from patients with multiple sclerosis (MS). Their putative capsid proteins share similarity to Torque teno virus (TTV) open reading frame 1 (ORF1) proteins.

## GENOME ANNOUNCEMENT

A large number of circular single-stranded DNA (ssDNA) viruses were recently identified by metagenomic analyses (1). The group of gemycircularviruses has been isolated from fungi, the feces of various animals, and plant leaves (2, 3). We report here the isolation of the genome sequences of 3 novel viruses distantly related to this virus group.

We analyzed blood samples taken from healthy cattle and serum samples from patients suffering from multiple sclerosis. The bovine sera were subjected to density gradient centrifugation, followed by rolling circle amplification (RCA) on DNA from single fractions. The fragments obtained after restriction digestion (BamH1 and EcoR1) were cloned into vector pUC19 and sequenced by primer walking (4). Both enzymes restricted HCBI8.215 (HCBI, healthy cattle blood isolate) (2,152 bp) and the isolate from human serum, MSSI2.225 (multiple sclerosis serum isolate) (2,259 bp), and both fragments of each were cloned and sequenced. HCBI9.212 (2,121 bp) was isolated from 2 different serum pools, and the complete genome was verified by inverted PCR amplification. The primers used were forward primer 5′-CCGGAATTCCTCCATCCGAAT-3′ and reverse primer 5′-CCGGAATTCATTACCACATATAT-3′. We subsequently isolated a sequence, that of MSBI3.224 (MSBI, multiple sclerosis brain isolate), which was identical to that of MSSI2.225, from postmortem multiple sclerosis (MS)-affected brain tissue.

The genome organization of all 3 isolates revealed a putative spliced replication protein encoded on the negative strand and the coat protein (CP) on the positive strand. The CP was arginine rich, and a similarity to the Torque teno virus (TTV) open reading frame 1 (ORF1) protein was indicated in a DomainSweep analysis (5). The putative rolling circle motifs I, II, and III and a Walker B motif for each were identified as follows: for HCBI8.215, LLTYA, HLHAFVD, YAIKD, and VFDDI; for HCBI9.212, LLKMP, HYHIYLG, YVGKD, and VFDDI; and for MSSI2.225, LLTYP, HLHAFVD, YAIKD, and IFDDF, respectively. The GRS motifs were AVFDVGGFHPNISITK, TAFDYFGAHGNIKSIR, and RAFDVEGCHPNVSPSR for the three isolates, respectively. The nonanucleotide motif for both HCBI8.215 and MSSI2.225 is TAATGTTAT, and for HCBI9.212, it is TAATATTAT.

The isolation of these 3 gemycircularvirus-related genomes directly from animal and diseased human tissue poses the question of whether they have some etiological relevance for diseases, such as multiple sclerosis.

### Nucleotide sequence accession numbers.

The complete sequences of HCBI8.215, HCBI9.212, and MSSI2.225 have been deposited in the EMBL Databank under accession numbers LK931483, LK931484, and LK931485, respectively.
